# Cytotoxicity and antifungal effects of combined dexamethasone and miconazole on human oral keratinocytes, gingival fibroblasts, and *Candida albicans*

**DOI:** 10.1007/s10266-025-01119-z

**Published:** 2025-05-21

**Authors:** Papon Muangsanit, Supathep Tansirichaiya, Puangwan Lapthanasupkul, Chidchanok Leethanakul, Kununya Pimolbutr, Weerachai Singhatanadgit

**Affiliations:** 1 Cell Technology and Tissue Engineering Research Team, National Center for Genetic Engineering and Biotechnology (BIOTEC), National Science and Technology Development Agency (NSTDA), Pathumthani, Thailand; 2 Department of Microbiology, Faculty of Medicine Siriraj Hospital, Mahidol University, Bangkok, Thailand; 3 Department of Oral and Maxillofacial Pathology, Faculty of Dentistry, Mahidol University, Bangkok, Thailand; 4 Department of Preventive Dentistry, Faculty of Dentistry, Prince of Songkla University, Songkhla, Thailand; 5 Department of Oral Medicine and Periodontology, Faculty of Dentistry, Mahidol University, Bangkok, Thailand; 6 Research Unit in Mineralized Tissue Reconstruction and Faculty of Dentistry, Thammasat University, Khlong Luang, Pathum Thani, Thailand

**Keywords:** Dexamethasone, Miconazole, *Candida albicans*, Cytotoxicity, Oral lichen planus

## Abstract

Oral lichen planus (OLP), a prevalent immune-mediated inflammatory condition, requires effective therapies. Topical corticosteroids, such as dexamethasone, are widely used for OLP treatment. However, they can predispose patients to secondary candidiasis, necessitating adjunctive therapy with antifungal agents like miconazole. Little is known about the cellular dynamics and toxicity of the combined use of dexamethasone and miconazole. This study examined the effect of dexamethasone on the antifungal activity of miconazole against *Candida albicans* and the effect of miconazole on the immunosuppressive activity of dexamethasone on human T cells. The cytotoxicity of dexamethasone alone and dexamethasone combined with miconazole on human oral keratinocytes and gingival fibroblasts was also determined using both in vitro monolayer and Transwell co-culture models. A 5-min incubation thrice daily cell treatment protocol was employed for all assays. Dexamethasone did not affect miconazole’s antifungal efficacy, and a single exposure of miconazole inhibited over 99% of *C. albicans* growth. In monolayer cultures, 0.05% dexamethasone was non-toxic to keratinocytes and fibroblasts, while miconazole exhibited dose-dependent cytotoxicity at high concentrations. Transwell co-culture models confirmed this dose-dependent cytotoxicity, with higher miconazole concentrations causing increased apoptosis. Dexamethasone significantly reduced T cell viability, activation, and proliferation, unaffected by miconazole co-treatment. In conclusion, when used in combination at optimal concentrations, miconazole’s antifungal activity and dexamethasone’s anti-T-cell proliferation activity are retained without cytotoxicity to human oral cells. Further research is needed to validate these findings and develop evidence-based treatments for oral lichen planus.

## Introduction

Oral lichen planus (OLP) is one of the most common chronic immune-mediated inflammatory conditions, affecting approximately 1% of the world’s population. It predominantly occurs in middle-aged and elderly females [1]. The etiology of OLP remains unclear. Clinically, OLP can manifest with a wide range of clinical features from asymptomatic white reticular striation to painful ulcerative lesions with fluctuation in severity over time [2]. Diagnosis of OLP is based on both clinical and histological examination. Histopathology in patients with OLP is characterized by T-cell inflammatory infiltration (CD4^+^ and CD8^+^ T lymphocytes) along with hyperkeratosis, atrophic epithelium, and basal cell degeneration [3].

The exact pathogenesis of OLP is unclear. Current evidence suggests that OLP reflects a dysregulation of T cell-mediated immune mechanisms resulting in the degeneration of basal cell keratinocytes. An immune response may be triggered by unknown antigens. This process is thought to be mediated by antigen-presenting cells, mainly Langerhans cells, or oral keratinocytes presenting antigen to CD4 + T-cells. The activated CD4 + T-cells then secrete interleukin-2 (IL-2), interleukin-12 (IL-12), interferon-gamma (IFN-γ) and activate CD8 + T-cells resulting in keratinocyte apoptosis which contributes to basal cell degeneration in OLP pathology [4].

For decades, topical corticosteroids remain the mainstay in the management of immune-mediated oral mucosal diseases (i.e., OLP, pemphigus vulgaris, mucous membrane pemphigoid) as they have anti-inflammatory and immunosuppressive properties. Systemic corticosteroids are usually reserved for acute exacerbation, recalcitrant or severe erosive lesions due to their adverse effects [5, 6]. Topical corticosteroids are available in a diverse range of potencies ranging from low potency to high potency and in several different preparations including sprays, mouthwashes, oral paste, gels, ointments, and mucoadhesive oral patches [5]. Several topical corticosteroids such as triamcinolone acetonide, fluocinonide, clobetasol propionate, fluticasone, betamethasone as well as dexamethasone (Dex) have been investigated and shown to be effective in the treatment of chronic oral mucosal diseases including OLP [6, 7]. However, there remains no standard recommendation on which formulation, steroid potency should be used as well as the frequency of applications [7]. This is due to a lack of well-designed and sufficiently powered clinical trials. Data concerning the local toxicity and effective doses of a steroid derived from studies mimicking practical treatment frequency, i.e., 3 times daily, are essential for informing the design of forthcoming preclinical and clinical investigations. However, all current data are derived from experiments involving the continuous exposure of culture models to a drug, which provides clinically irrelevant outcomes.

Oral mucosal diseases, like OLP, may be associated with superimposed candidal infection, with *Candida albicans* being the most prevalent [8, 9]. In addition, long-term use of topical and/or systemic corticosteroids can predispose patients to secondary candidiasis [10]. Infections caused by *Candida* species can lead to both exacerbation and obscuration of the clinical features of OLP, as both conditions could be associated with burning pain and erythematous lesions [11]. The treatment of OLP can be challenging in the presence or development of *Candida* superinfection, therefore antimycotic therapy is necessary to be supplemented in combination with corticosteroids. Miconazole (Mz), an azole antifungal, has successfully been used to treat secondary oral candidiasis in patients receiving immunosuppressants [12, 13]. It elicits antifungal activity via inhibition of ergosterol synthesis, a primary component of fungal cell membranes [12]. It also exhibits strong inhibitory activity against several *Candida* strains including *C. albicans* [14]. Carbone et al*.* (1999) demonstrated the use of concomitant antimycotic agents with Mz gel together with chlorhexidine mouthwashes seemed to be useful in preventing oral candidiasis among OLP patients being treated with topical corticosteroids [15]. In another study by Lodi et al., the concurrent use of Mz gel and clobetasol propionate was found to be effective in preventing the occurrence of secondary oral candidiasis in OLP patients. OLP patients who received clobetasol propionate gel twice daily and Mz once daily showed no clinical candidiasis infection compared to the placebo controls [16]. Despite several clinical uses of antifungal drugs as an adjuvant treatment of topical corticosteroids, there remains a lack of clinical studies regarding the use of Mz combined with Dex. In addition, Mz has been shown to inhibit glucocorticoid receptor functions, resulting in a decreased expression of cytochrome P450 3A4 (CYP3A4) gene which mainly governs drug metabolism including Dex [17]. Thus, local intraoral co-application of Dex and Mz may influence their expected effectiveness. However, no studies have investigated the interaction between Dex and Mz in oral cells and their combined efficacy.

This study aimed to investigate the antifungal activity of Mz against *C. albicans* and immunosuppressive activity against T cells of Dex using the dual drug treatment. The effects of Dex alone and Dex supplemented with Mz treatments on the viability of human normal oral keratinocytes (NOKs) and gingival fibroblasts (GFs) in both in vitro monolayer and Transwell co-culture models were also examined. All assays followed a treatment protocol involving 5-min incubations applied three times daily. Insights gained from this research will contribute to the development of evidence-based treatment approaches, fostering precision and efficacy in addressing the multifaceted challenges posed by OLP.

## Material and methods

### Biosafety and ethical approval

The biosafety of the protocols of this study was approved by the Safety and Risk Management Taskforce Mahidol University (SI2023-006) and the Institutional Biosafety Committee Thammasat University (034/2564). Ethical approval for the isolation and use of human primary cells in this study was approved by the Ethics Review Sub-Committee for Research Involving Human Research Subjects of Thammasat University No. 3 (049/2564).

### Antifungal susceptibility assay

The minimum inhibitory concentration (MIC) of Mz against *C. albicans* ATCC 24433 was determined according to the Clinical and Laboratory Standards Institute (CLSI) M27 standard [18]. This was performed as a baseline control for the antifungal efficacy of Mz. The medium used in this study was RPMI-1640 broth with glutamine, without bicarbonate and buffered to pH 7.0 with MOPS. A *C. albicans* stock suspension was prepared by selecting five colonies of *C. albicans* grown on yeast extract–peptone–dextrose (YPD) agar, subculturing into 5 mL of 0.85% saline, and adjusting to 0.5 McFarland standard at 530 nm wavelength.

For the 24-h microbroth dilution assay, *C. albicans* working suspension was made by preparing a 1:1000 dilution of the stock suspension in RPMI-1640 broth. Mz working solution was prepared with concentrations ranging from 0.015625–2 µg/ml in RPMI-1640 broth, and 0.05% Dex was added for the Mz + Dex group. The broth microdilution was performed by adding 50 µl of each miconazole working solution and 50 µl *C. albicans* working suspension. The plates were incubated at 37 °C for 24 h and checked for growth to determine the MIC.

For the 3-time exposure assay, a 1:50 dilution of the *C. albicans* was prepared in RPMI-1640 broth. Miconazole working solution was prepared with concentrations ranging from 0.25–64 µg/ml in RPMI-1640 broth. One milliliter of the diluted cell suspension was aliquoted into each Eppendorf tube and centrifuged to pellet the cells. The supernatant was removed, and the resuspended cells were incubated with different concentrations of Mz or Mz + Dex working solution. All tubes were incubated at 30 °C with shaking at 200 rpm for 5 min, then washed and resuspended the cells in fresh RPMI-1640 broth. Cells were then incubated at 30 °C without shaking for 4 h, repeated the same process twice, and then incubated all tubes at 30 °C for 18 h. Cells were diluted in a normal saline solution and spread on YPD agar. The number of colonies on each plate was counted after incubation at 37 °C for 2 days. The percentage of inhibition was calculated by dividing the number of colonies from each concentration by the number of colonies in the control plate.

### Culture of human NOKs, GFs, and T lymphocytes

NOKs are spontaneously immortalized normal oral keratinocytes isolated from gingival tissues of healthy volunteers [19]. NOKs were maintained in a defined keratinocyte serum-free medium (KSFM: Gibco Life Technologies Ltd, Paisley, UK) containing 1% penicillin and streptomycin (both from Gibco) at 37 °C in a humidified atmosphere of 5% CO_2_ in the air. The medium was changed every 2–3 days. These cells have previously been reported to have characteristics resembling normal oral keratinocytes [19].

GFs were isolated from biopsies of healthy patients’ gingiva. They were cultured in Dulbecco’s modified Eagle medium (DMEM) containing 10% fetal calf serum (FCS) supplemented with 1% penicillin and streptomycin and 2 mM L-glutamine (all from Gibco) at 37 °C in a humidified atmosphere of 5% CO_2_ in the air. GFs between passages 3–5 were used.

T lymphocytes were isolated from buffy coats of fully anonymized healthy donors who provided blood donations to the Thammasat University Hospital Blood Bank. The diluted buffy coat was layered on Ficoll-Paque PLUS (GE Healthcare Life Sciences) and centrifuged at 800 g for 40 min at 18 °C. The peripheral blood mononuclear cell (PBMC) interface layer was collected, washed three times with phosphate-buffered saline (PBS), and incubated in the RPMI medium containing 10% FCS supplemented with 1% penicillin and streptomycin and 2 mM L-glutamine (all from Gibco) for 2 h. The non-adherent T cells were collected and activated with 5 µg/ml concanavalin A (ConA) for 72 h before use.

### Keratinocyte-fibroblast Transwell co-culture model

**Fig. 1:**
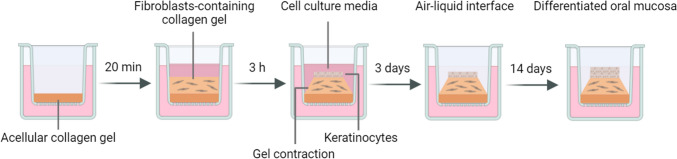
Schematic diagram showing the fabrication of NOKs-GFs Transwell co-culture model

The Transwell co-culture models of the oral mucosa were fabricated by preparation of the collagen mixture with type 1 rat tail collagen (3 mg/ml in 0.6% acetic acid; Gibco), 10 × minimum essential medium (Sigma-Aldrich), and neutralized with 1 N sodium hydroxide before an addition of GF suspension. 1.5 mg/ml collagen mixture was prepared with 2.7 × 10^6^ GFs in 150 µl final volume. 50 µl of acellular 1.5 mg/ml collagen hydrogel was first added to the Transwell insert of 24-well insert (6.5 mm diameter with 0.4 µm pore size), and then followed by fibroblasts-containing collagen hydrogels. The hydrogels were incubated at 37 °C, 5% CO_2_ for 2 h to polymerize and for another 1 h after adding fibroblast media. Finally, 1 × 10^5^ NOKs were seeded on top of the GFs-populated collagen hydrogels, and the construct was incubated and submerged in KSFM for 3 days. After 3 days, the insert was raised to an air–liquid interface for 14 days in the differentiation media (KSFM + 1.2 mM CaCl_2_ + 0.25 mmol/l ascorbic acid). The culture medium was changed every 2 days. The procedures are presented in Fig. 1.

### Drug treatment

For the cell monolayers, cells were treated with 0.05% Dex in the presence of Mz at 0, 4, 6, 8, and 64 μg/ml [16, 20], following the clinical regimen recommended for patients with OLP, i.e., 5 min for 3 times per 24 h [21]. After 48 h, cells were assayed as indicated below. Non-treated cells and cells treated with Dex alone were used as controls.

For the Transwell co-culture model, drugs were diluted in 150 µl KSFM and added into the insert. Drugs were added to the co-culture models 3 times daily, treated every 4 h apart, for 5 min each at 37 °C, 5% CO_2_. The models were washed with fresh KSFM prior to each drug treatment. Fresh KSFM without drugs was used in the control group. This drug treatment was continued for 2 days before the samples were fixed in 4% paraformaldehyde (PFA) overnight at 4 °C, washed with PBS, and then stored at 4 °C until analysis.

### Morphological analysis and cell viability assay of NOKs and GFs

Cells were cultured at a density of 5 × 10^3^ cells/cm^2^ for 18 h. After the indicated time in culture, the cell morphology was examined under a light microscope (Nikon Eclipse TS100), and photomicrographs of cell appearances were taken using a Nikon Digital sight DS-L2. Cells were then fixed with 4% PFA for 15 min and stained with 0.05% crystal violet solution for 15 min. The cell morphology was examined under a light microscope, and photomicrographs of cell appearances were taken as above. For the cell viability assay, 3-(4,5-dimethylthiazol-2-yl)-2,5-diphenyltetrazolium bromide (MTT) assay was performed using 0.2% MTT solution for 4 h at 37 °C, and the end-product color was subsequently analyzed by measuring absorbance at 490 nm (A_490_) which corresponds to the viability of cells. Cell viability is expressed as the mean percentage ± SD of control (100%) from three independent experiments.

### Histological assessment and immunohistochemistry of Transwell co-culture model

The NOKs-GFs Transwell co-culture models were fixed with 4% PFA overnight at 4 °C. The organ cultures were submitted for routine tissue processing in the automatic tissue processor. First, the specimens were dehydrated through an alcohol series ranging from 70% to absolute and then cleared with xylene. Subsequently, the specimens were embedded in paraffin wax and the tissue blocks were sectioned using a microtome to produce 3-μm slices. Finally, the sections were stained with hematoxylin and eosin (H&E) and observed under a light microscope.

Immunohistochemistry (IHC) was performed on 3-µm thick sections with primary antibody against Ki67 (Ki67 mouse monoclonal antibody, dilution 1:300, Dako, Carpinteria, CA, USA) and E-cadherin (E-cadherin mouse monoclonal antibody, dilution 1:150, Novocastra, Leica Biosystems, Newcastle Upon Tyne, UK) for 40 min on the LEICA BOND-III IHC automated stainer (Leica Biosystems). Before mounting the slides, they were counterstained with hematoxylin for 20 min. To support the validity of the staining, positive controls for Ki67 (Appendix tissue) and E-cadherin (Breast carcinoma) were also included in each run. ImageJ software was used to count the number of Ki67-positive cells (4–5 regions per condition).

### TUNEL assay

To assess the apoptotic cells of the Transwell co-culture models, 3-µm thick sections were subjected to indirect TUNEL staining using the ApopTag® Plus Peroxidase In Situ Apoptosis Detection Kit (S7101, Sigma-Aldrich). Micrographs of the NOKs-GFs Transwell co-culture model were captured using a microscope (Olympus). ImageJ software was used to count the number of TUNEL-positive cells (4–5 regions per condition).

### Analysis of T-cell viability, activation, and proliferation

The viability, activation, and proliferation of T cells in each sample were assessed to determine the effect of Dex in the presence and absence of Mz. T cells were labeled with 1 μM 5-(and 6)-carboxyfluorescein diacetate succinimidyl ester (CSFE) (eBioscience™) and CFSE-labeled and non-labeled cells were treated with drugs as described above, with and without 5 μg/ml ConA activation before subjected to propidium iodide staining (for non-viable cell detection) and flow cytometry using the CytoFLEX Flow Cytometer (Beckman Coulter, CA, USA). The acquisition data were analyzed using the ModFit LT 6.0 software (Verity Software House, USA). Propidium iodide-positive cells were considered non-viable cells. Activated and non-activated T-cell populations were gated from propidium iodide-negative cells, with T cells cultured without ConA stimulation being used as a reference for non-activated cells in the flow cytometric dot plots. The proliferation index (PI) was analyzed using the ModFit LT 6.0 software, which automatically calculated the PI of T cells as the sum of cells in all generations divided by the number of original parent cells. The proportion of divided T cells was also obtained from the software.

### Statistical analysis

All statistical analyses were conducted using GraphPad Prism version 9.0.0 (GraphPad Software, Boston, MA) and STATA version 16.0 software (StataCorp LLC, College Station, Texas). The data normality was assessed using the Shapiro–Wilk test prior to conducting one-way ANOVA. One-way ANOVA with Bonferroni’s post hoc test was performed to determine statistically significant differences between multiple groups. A *p* value < 0.05 was considered as statistically significant.

## Results

### Effects of Dex on antifungal activity of Mz against *C. albicans*

**Fig. 2:**
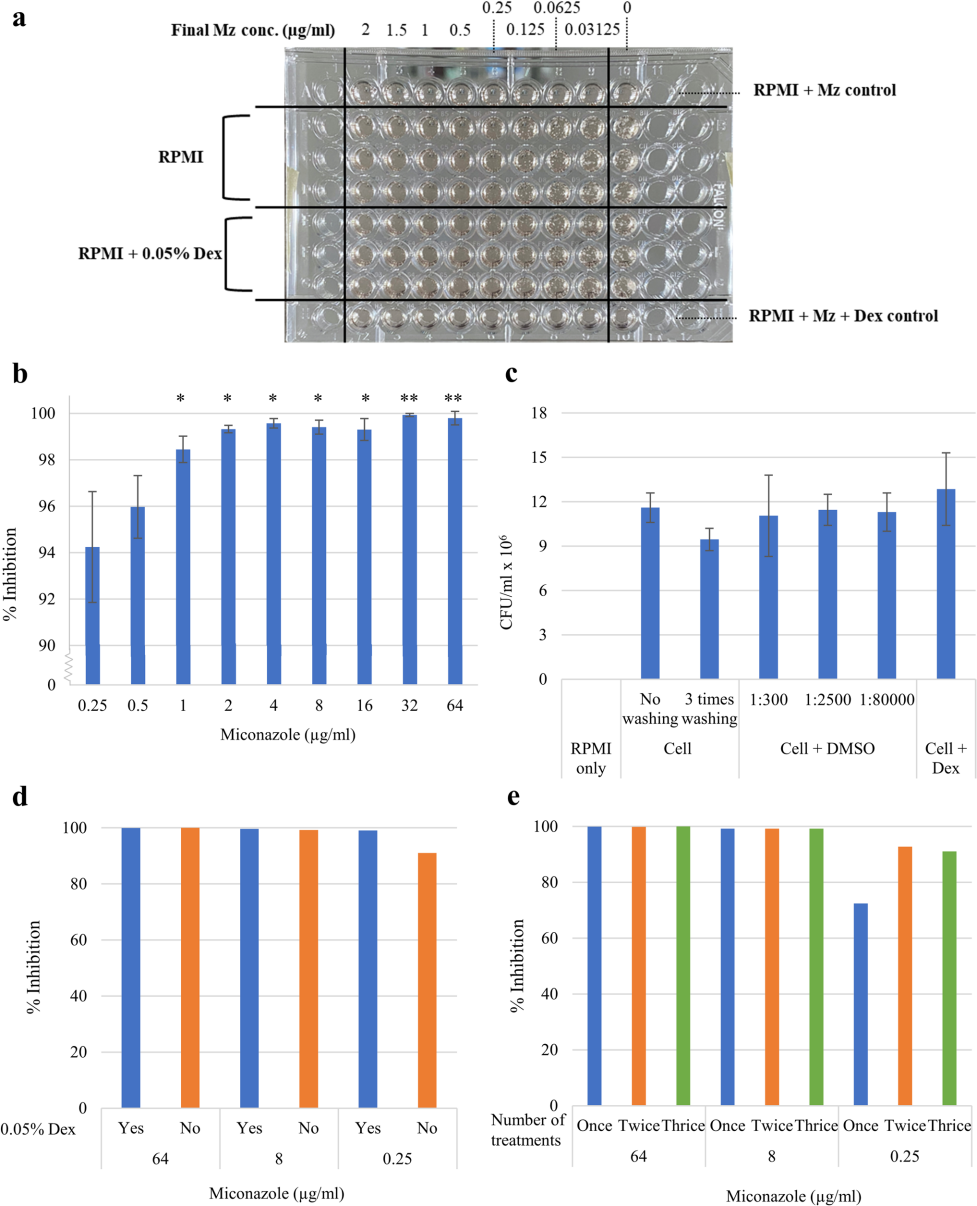
Antifungal activity of miconazole against *C. albicans*. **a** The effects of Dex on the antifungal activity of Mz were determined in 96-well microtiter plates after 24 h incubation in three biological replicates. **b-e** The inhibition of *C. albicans* exposed to a 5-min Mz treatment protocol. The percentage of inhibition on *C. albicans* and the number of cells in the control groups are shown in **b** and **c**, respectively. The effects of Dex and the number of Mz treatment are shown in **d** and **e**, respectively. The results shown in **b** and **c** are presented as the mean percentage ± SD from three independent experiments. n = 3. Statistical significance is indicated by *p < 0.05 vs % inhibition at 0.25 µg/ml, and **p < 0.05 vs % inhibition at 0.5 µg/ml

The effects of Dex on the antifungal efficacy of Mz against *C. albicans* were assessed by means of a 24-h broth microdilution assay performed in 96-well microtiter plates. The MIC of Mz against *C. albicans* was determined from three independent biological replicates for both the Mz-only and Mz + Dex groups and was found to be equivalent at 0.5 µg/ml (as shown in Fig. 2a).

### Determine of the MIC of Mz to inhibit *C. albicans* from 3-time exposure

The concentration of Mz that could inhibit *C. albicans* after being exposed to the agent 3 times for 5 min was then determined. Results showed that treating *C. albicans* with Mz 3 times per day with concentrations between 0.25 and 64 µg/ml resulted in an inhibition ranging from 94.24 to 99.93% (Fig. 2b). Statistically significant differences were observed in the percentage of inhibition at 0.25 µg/ml Mz compared to 1–64 µg/ml Mz, as well as at 0.5 µg/ml Mz compared to 32–64 µg/ml Mz. Control groups showed no statistically significant difference in CFU among each condition (Fig. 2c), suggesting that DMSO (the solvent of Mz) and Dex did not affect the growth of *C. albicans*.

Further experiments were conducted to assess the effect of adding 0.05% Dex on the inhibitory activity of Mz. Results showed that the inhibitory activity of Mz was not lowered by Dex, as evidenced by a similar inhibition effect in the groups added with 8 and 64 µg/ml Mz (Fig. 2d). However, the inhibitory activity was increased by 8% when 0.05% Dex was added to the group containing 0.25 µg/ml Mz. In addition, the effects of the number of Mz treatments were tested at 0.25, 8, and 64 µg/ml, which showed that treating *C. albicans* at 8 and 64 µg/ml once for 5 min was enough to inhibit more than 99% of *C. albicans*, while the 0.25 µg/ml needed to be treated twice to have more than 90% inhibition (Fig. 2e).

### Effect of drug treatment on the viability of GFs and NOKs

**Fig. 3:**
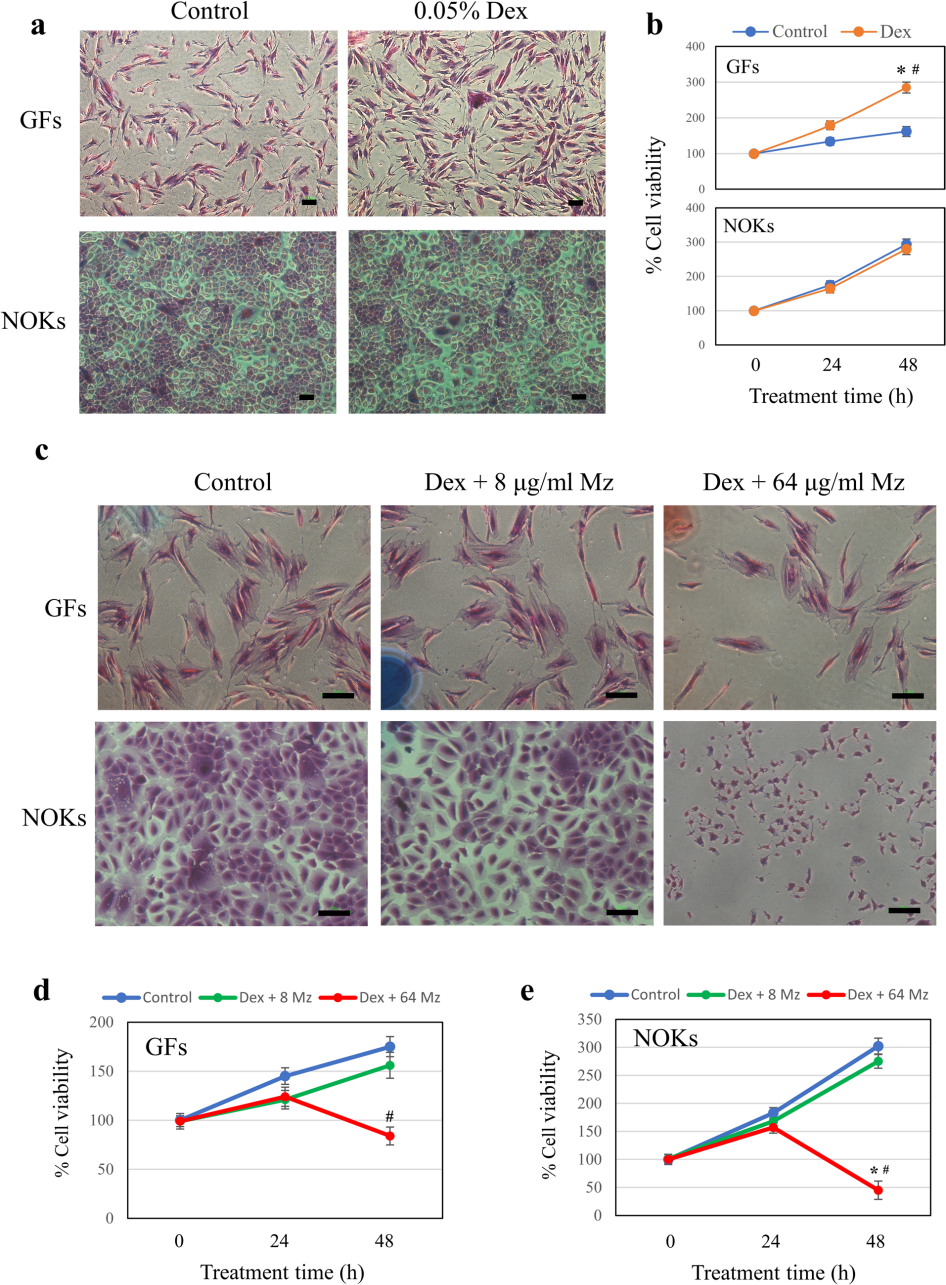
Effect of Dex and Mz on the viability of GFs and NOKs. Cells were exposed to a 5-min drug treatment protocol (3 times/24 h) for 48 h. Dex at 0.05% and Mz at 8 and 64 µg/ml were used. The treated cells were stained with crystal violet (**a**, **c**), and the viability was measured by MTT (**b–e**). The results shown in **b**, **d**, and **e** are presented as the mean percentage ± SD from three independent experiments. n = 3. *p < 0.05 vs control cells at 0 h; #*p* < 0.05 vs control cells at 48 h. Scale bar = 100 µm

The effect of a clinically relevant regimen of Dex (at 0.05% 3 times per day) on the viability of GFs and NOKs was first determined. Dex at this concentration appeared to increase the number of GFs while it had no discernible effects on the number of NOKs (Fig. 3a). Morphologically, both cell types treated with Dex were spindle-to-oval and polyhedral, respectively, comparable to their control cells. In Fig. 3b, MTT results confirmed that Dex increased GFs by 130% and 170% at 24 h and 48 h, respectively, after treatment. Co-treatment with Dex and 64 μg/ml Mz for 48 h, but not 24 h, was cytotoxic to both GFs and NOKs, reducing the number of morphologically normal cells and decreasing cell viability by 2- and 7.5-fold, respectively (Fig. 3c–e). In contrast, co-treatment with 8 μg/ml Mz, which strongly suppressed *C. albicans,* had little to no effect on the viability of both cell types at both time points (Fig. 3c–e). This suggests that Mz concentrations not exceeding 8 μg/ml appeared to be non-toxic when co-administered with a clinically used concentration of Dex.

### Effect of drug treatment on the keratinocyte-fibroblast Transwell co-culture model

**Fig. 4:**
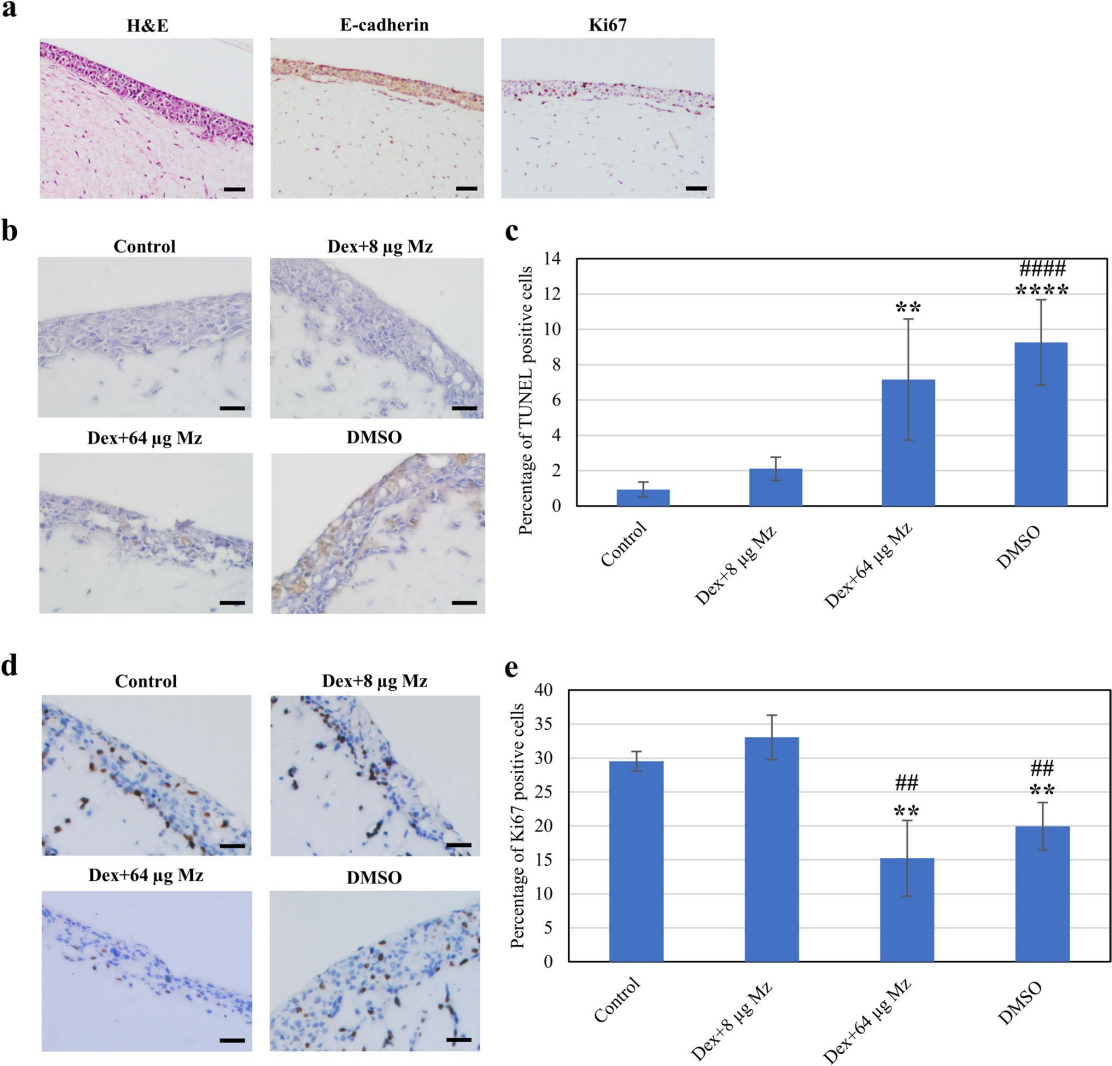
Effect of Dex and Mz on the viability of oral keratinocyte-fibroblast Transwell co-culture model. The NOK-GF Transwell co-culture models were fabricated and morphologically characterized via H&E, E-cadherin and Ki-67 staining (**a**). The Transwell co-culture models were exposed to a 5-min drug treatment protocol (3 times/24 h) for 48 h. Dex at 0.05% and Mz at 8 and 64 µg/ml were used. Drug cytotoxicity was analyzed by staining with TUNEL staining assay kit (**b**), and the percentage of TUNEL-positive cells per field of view was calculated by counting manually using ImageJ (**c**). Cell proliferation was also analyzed by staining with Ki-67 (**d**), and the percentage of Ki-67 positive cells per field of view was calculated by counting manually using ImageJ (**e**). The results are presented as the mean ± SD from five analyzed images from two independent experiments. *n* = 5. Statistical significance is indicated by ****p < 0.0001, **p < 0.01 vs control group; ####*p* < 0.0001, ##*p* < 0.01 vs Dex + 8 µg/ml Mz. Scale bars = 100 µm

Here, the toxicity of Dex and Mz in an in vivo-like keratinocyte-fibroblast Transwell co-culture model was also examined, using NOKs and GFs. H&E staining showed that the NOKs and GFs co-culture within the collagen hydrogel Transwell model can be generated (Fig. 4a). GFs homogeneously populated the collagen hydrogels and NOKs formed layers on top. Despite the thin layers, NOKs expressed E-cadherin, a tight junction marker, at the top layer, and Ki-67-positive cells confirmed epithelial cell proliferation in our model.

The Transwell co-culture model was used to evaluate drug toxicity over 2 days with three treatments to mimic a mouthwashing pattern. A higher concentration of Mz (64 µg/ml) significantly increased apoptosis compared to the lower dose (8 µg/ml) (Fig. 4b, c). Furthermore, the highest dose and the DMSO group showed a reduced percentage of proliferating cells compared to the control (Fig. 4d, e). Overall, the results from the Transwell model aligned with the two-dimensional (2D) monolayer model, suggesting 8 µg/ml Mz with 0.05% Dex as the optimal dose.

### Effect of drug treatment on T-cell viability, activation, and proliferation

**Fig. 5:**
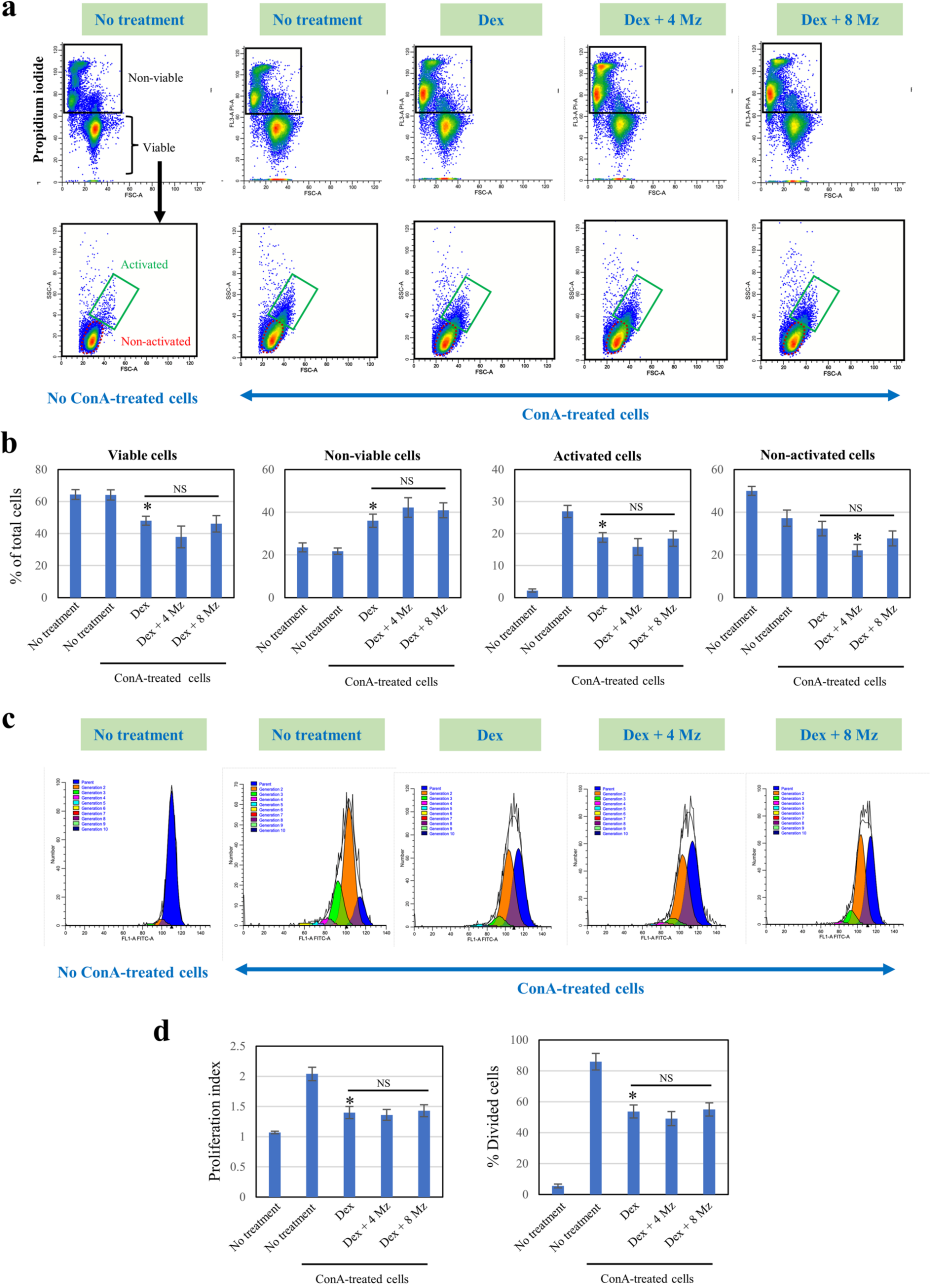
Effect of Dex and Mz on the viability, activation, and proliferation of T lymphocytes. ConA-stimulated T cells pre-labeled with CFSE were exposed to a 5-min drug treatment protocol (3 times/24 h) for 48 h. Dex at 0.05% and Mz at 4 and 8 µg/ml were used. The cell samples were stained with propidium iodide, and the cell viability and activation (**a**, **b**) and proliferation (**c**, **d**) were analyzed by flow cytometry. The results are presented as the mean ± SD from three independent experiments. *n* = 3. *p < 0.05 vs ConA-activated cells

Based on its cytocompatibility to both GFs and NOKs, Mz at concentrations of 4 and 8 μg/ml was selected to determine its influence on the effect of Dex on T-cell viability, activation, and proliferation. In Fig. 5a–b, Dex treatment significantly reduced T-cell viability, causing an almost twofold increase in non-viable T cells, and also significantly decreased the number of activated T cells from about 27% to 18%. Co-treatment with Mz at both concentrations did not alter the suppressive effect of Dex on the number of viable T cells and activated T cells. Dex did not significantly reduce the number of non-activated T cells, whereas co-treatment with 4 μg/ml Mz significantly lowered their number (Fig. 5b). In Fig. 5c and d, the proliferation index and the proportion of divided cells in T cells treated with Dex were significantly reduced to 1.42 (vs 2.04 in the untreated T cells) and 54% (vs 86% in the untreated T cells), respectively. Mz at these concentrations did not affect Dex-suppressed T-cell proliferation. The results demonstrated that the Dex treatment protocol used in this study reduced the viability, activation, and proliferation of T cells, and these suppressive effects of Dex were not influenced by co-treatment with Mz (at 4 and 8 μg/ml).

## Discussion

This is the first study to investigate the impact of Dex on the antifungal activity of Mz against *C. albicans* and the effect of Mz on the immunosuppressive activity of Dex on human T cells in the context of OLP treatment. This study also explored the cytotoxic effects of Dex alone and in combination with Mz on human oral cells (NOKs and GFs). The study aimed to assess the potential impact of these agents on antifungal activity, cell viability, and immune responses, utilizing both in vitro monolayer and Transwell co-culture models. Drug treatment protocols tested in several previous studies are not clinically relevant, i.e., continuous exposure of cells to drugs for hours to days, which might not accurately reflect real clinical practice [22–24]. Clinically, in the treatment of OLP, steroid or immunosuppression mouthwashes are recommended to be used for at least 3–5 min up to two or three times daily [21, 25–27]. To better replicate real-world application, this study implemented a 5-min incubation repeated three times a day. This marks the first assessment of antifungal activity, immunosuppressive effects, and cytotoxicity under this clinically relevant regimen, providing novel insights into treatment dynamics.

In the monolayer cell cultures, 0.05% Dex treatment 3 times/day for 2 days appeared to be non-toxic to both GFs and NOKs, two major cell populations involved in OLP lesions. Our findings indicated that a high concentration of Mz (64 µg/ml), but not a lower concentration (8 µg/ml), was detrimental to both NOKs and GFs, suggesting a dose-dependent response. Lam et al*.* (2022) showed that the cytotoxicity of Mz on HaCaT keratinocytes was dose-dependent and involved excessive reactive oxygen species generation as observed at a concentration of 12.5 µg/ml after 24 h of exposure in vitro [23]. Not many studies have examined the cytotoxicity of Dex in oral cells. Tamari et al*.* (2019) reported that 10 µM of Dex (0.00039%) did not cause any significant toxicity to primary adult human GFs and HaCaT keratinocytes after 72 h in culture [28]. In addition, Li et al*.* (2022) examined the cytotoxicity of Dex-loaded nanocomposite hydrogels on human GFs at day 1 and day 3 in vitro, aiming to develop treatments for periodontal disease [29]. Numerous studies utilized 2D monolayer cultures of keratinocytes and fibroblasts to study the cytotoxicity of antifungal and antimicrobial drugs [23, 30]. However, these culture models do not recapitulate the three-dimensional (3D) architecture of the native oral mucosa hindering clinical translation. A previous study demonstrated that the antifungal silver-containing microcrystals did not affect the proliferation and metabolic activity of the human GFs using 3D collagen hydrogel models, whereas the silver-containing microcrystals exhibited a detrimental effect on the 2D culture of GFs emphasizing the importance of more complex culture models [31]. Therefore, the present study also employed the co-culture Transwell model of human NOKs and human GFs-embedded collagen hydrogels to evaluate drug toxicity. The concentration of 0.05% Dex combined with Mz also showed similar results in the co-culture Transwell model. Higher concentrations of Mz were associated with increased apoptotic cells, indicating a potential dose-dependent cytotoxic effect, supporting the findings from the 2D monolayer cultures. Whether the drugs affect the oral epithelium barrier integrity is another aspect worth exploring further.

T cells play a vital role in the pathogenesis of OLP, particularly in mediating the autoimmune nature of the disease leading to the disruption of mucous membrane of the oral cavity. Treatments of OLP, therefore, predominantly aim to reduce inflammation by modulating this T-cell-mediated immune response. Dex is widely used to treat OLP owing to its anti-inflammatory effect and T-cell suppression capability [32]. Chen *et* al. (2018) investigated the effects of different doses of Dex (0.1–100 µg) on the viability and activity of natural killer (NK) cells and T cells in mice models that were injected with Dex for 3 consecutive days [22]. They found that Dex suppressed the activity of NK cells in the spleen and exhibited a dose-dependent decrease in both CD4 + and CD8 + T cells. In our study, the combination of 0.05% Dex and Mz at various concentrations was shown to significantly reduce T cell viability, activation, and proliferation. This suggests that Mz did not interfere with the efficacy of Dex on T cell activity, implying that Dex combined with Mz could be an effective treatment for OLP patients.

The antifungal susceptibility assays indicated that the MIC of Mz against *C. albicans* was not significantly affected by the addition of Dex. The 3-time exposure assay showed the ability of Mz to inhibit *C. albicans*, showing no significant interference when combined with Dex. Dex also exhibited a potential synergistic effect with Mz, as evidenced by an 8% enhancement in the inhibition of *C. albicans* when 0.05% Dex was combined with 0.25 µg/ml Mz in the 3-time exposure assay. This observation has been replicated in other studies which demonstrated that fluconazole and Dex can synergistically inhibit resistant *C. albicans* both in vitro and in vivo [33]. It was shown that Dex inhibited the efflux of fluconazole through the downregulation of efflux genes and also reduced phospholipase activity of *C. albicans*.

Dex is primarily metabolized by CYP3A4 enzymes and is known to induce the expression of CYP3A4 gene [34, 35]. This drug, therefore, can be affected by CYP450 inhibitors such as Mz [34]. Varis et al*.* (2000) reported an approximately fourfold increased exposure of Dex in healthy human subjects administered with Dex and itraconazole [36]. On the other hand, Li et al*.* (2020) demonstrated that there was no interaction between Ketoconazole (an Azole antifungal) and Dex during 12-h incubation in HaCaT keratinocytes in vitro [37]. Mz is also reported to be an antagonist of glucocorticoid receptor (GR), thereby inhibiting GR-mediated activities [17]. However, reported interactions between Mz and Dex were not observed in our in vitro study, which included both monolayer cultures and Transwell co-culture models. These in vitro models represented only local drug interactions. This suggests that the previously reported Dex–Mz interaction may predominantly be mediated through systemic mechanisms. Further in vivo studies are needed to confirm this hypothesis.

Our in vitro findings suggest a potential therapeutic regimen for OLP patients particularly those with a history of recurrent secondary oral candidiasis. The use of a pure Dex solution twice daily alongside a Dex-Mz solution administered once daily could be considered to prevent secondary oral candidiasis. This recommendation is grounded in three key observations: (1) the combination of 0.05% Dex and a high level of Mz demonstrate cytotoxicity to human oral cells, (2) a single exposure to 8 μg/ml Mz is adequate for achieving over 99% inhibition of *C. albicans* growth for at least 24 h, and (3) the pure Dex solution promotes the growth and proliferation of human oral cells, particularly GFs. With this proposed treatment, the combined solution is expected to effectively control *C. albicans* growth, while human oral cells can proliferate during the other two daily administrations of pure Dex solutions. Concurrently, Dex can suppress the viability and proliferation of T cells, the most crucial therapeutic target for OLP treatment. This regimen aimed to balance antifungal efficacy with the viability and proliferative potential of clinically relevant human oral cells. However, these findings are preliminary and should be interpreted with caution given the controlled in vitro conditions of the study. Further preclinical work is warranted before integration of these findings into clinical practice.

A primary limitation of the present in vitro study is the requirement for substantial preclinical studies to translate these findings into clinical application. Although this limitation is inherent to in vitro studies, the current findings offer crucial information on the optimal initial concentrations and feasibility of combining Mz and Dex for the development of new treatment approaches for OLP. In addition, the impact of Mz co-treatment on Dex-suppressed T-cell cytokine secretion remains undetermined, emphasizing the need for further investigation into this area. Another limitation is the use of simplified in vitro testing models, which do not fully mimic the complex interactions between immune cells and oral tissues observed in OLP in vivo. Future studies employing more advanced 3D co-culture models will help facilitate the transition to preclinical studies.

In conclusion, this in vitro study has shown that dual treatment of 0.05% Dex and 8 μg/ml Mz (three times daily for 2 days) may effectively suppress T-cell viability, activation, and proliferation while maintaining the viability of human NOKs and GFs and exhibiting potent antifungal activity against *C. albicans*. Further preclinical studies are warranted to validate these findings. Such studies should investigate the effects of longer treatment durations beyond 2 days and examine the impact on distinct T-cell subtypes. These data would provide essential knowledge for developing evidence-based therapeutic approaches for OLP.

## Data Availability

The data from which the findings of this research are derived are available upon request.
